# Unique lipid cargoes in APOE4 human brain-derived extracellular vesicles recruit cell adhesion molecules and promote tauopathy in Alzheimer’s disease

**DOI:** 10.21203/rs.3.rs-6508552/v1

**Published:** 2025-05-26

**Authors:** Tsuneya Ikezu, Zhengrong Zhang, Kaiwen Yu, Hanmei Bao, Tadafumi Ikezu, Arun Reddy Ravula, Bridgette Melvin, Clara Scholes, Yang You, Justice Ellison, Takahisa Kanekiyo, Zbigniew Wszolek, Michael DeTure, Dennis Dickson, Xianlin Han, Junmin Peng, Seiko Ikezu

**Affiliations:** Mayo Clinic Florida; Mayo Clinic Florida; St. Jude Children’s Research Hospital; University of Texas Health Science Center at San Antonio; Mayo Clinic; Mayo Clinic Florida; Mayo Clinic Florida; Universit of Manchester; Mayo Clinic Floria; Mayo Clinic Florida; Mayo Clinic Jacksonville; Mayo Clinic College of Medicine; Department of Neuroscience, Mayo Clinic, Jacksonville, Florida; Mayo Clinic Florida; UT Health San Antonio; St Jude Children’s Research Hospital; Mayo Clinic Florida

**Keywords:** Alzheimer’ Disease, apolipoprotein E, extracellular vesicles, free fatty acids, humanized animal models, induced pluripotent stem cells, lipidomics, neural cell adhesion molecule 1, proteomics

## Abstract

Brain-derived extracellular vesicles (BDEVs) carry tau filaments and promote tau transmission in Alzheimer’s disease (AD). However, how APOE ε4 allele, a key genetic risk factor for AD, may change BDEV molecular structures thereby facilitate disease progression is poorly understood. Here we report comprehensive analyses of BDEVs isolated from human E3/3 and E4/4 AD brains with a biological multi-omics approach. E4/4 BDEVs significantly enhanced tau propagation in aged human *MAPT* (Tau) knock-in and *APP*^NL-G-F^: Tau double knock-in mouse brains *in vivo* and increased neuronal uptake and excitability in induced pluripotent stem cell-derived neurons (iNeurons) compared to E3/3 BDEVs *in vitro*. Notably, correlation analysis of BDEV-lipidome and proteome exhibited synergistic enrichment in unsaturated free fatty acid (FFA)18:2, a precursor of inflammatory w6 FFA, and neural cell adhesion molecule 1 (NCAM1). Treatment of iNeurons with FFA 18:2 induces NCAM1 expression, recruits tau into EVs and enhance their tau seeding activity, which is blocked by NCAM1 antibody *in vitro*. Finally, intracerebroventricular injection of NCAM1 antibody significantly alleviated pathological tau accumulation and glial inflammation in PS19 tauopathy mouse brains, which had previously been reported to exhibit increased level of 18:2 FFA. This highlights novel pathological mechanism in tau transfer mediated by E4/4 BDEVs and emphasizes strong therapeutic potential of targeting EV molecules in AD progression.

## Introduction

Extracellular vesicles (EVs) are nanoscale membrane-bound particles, ubiquitously secreted from any cell types and mediate intercellular communication by transferring proteins, lipids, and RNA^1,2^ or serve as carriers of pathogenic proteins^3^. A defining feature of EVs is their lipid bilayer, which is highly enriched in cholesterol, sphingolipids, and phospholipids, a unique composition that preserves vesicle structure and stability while also modulating selective cargo packaging, vesicle trafficking, and uptake by recipient cells^4^. Emerging evidence suggests that pathological tau spread intercellularly via EVs in brains. In AD, brain-derived extracellular vesicles (BDEVs) encapsulate and transport disease-associated proteins, including amyloid-b peptide (Aβ) and tau, reflecting the molecular landscape of brain pathology^5^. Our previous studies demonstrated that BDEVs from AD patients exhibit a greater capacity to propagate tau pathology than oligomeric or fibrillar tau^6^. Cryo-electron microscopy has identified sarkosyl-insoluble globular forms and fibrillar tau filaments within individual BDEVs from AD brains^7^, supporting their role in disease propagation. Beyond that, BDEVs from AD brain also exhibited lipid alterations, such as glycerophospholipid and sphingolipid, compared with healthy controls^8^. Recent genome-wide association studies (GWAS) have revealed lipid metabolism and endosomal pathways as key contributors to AD genetic risk^9^, yet the precise roles of lipids and EV pathways in AD disease progression remain poorly understood. **APOE, a lipid related AD risk gene, is strongly implicated in disease pathogenesis, with** the **APO**E**ε4 allele** significantly increasing late-onset AD risk and accelerating disease progression through lipid dysregulation, Aβ aggregation, and exacerbate tau pathology^10,11^. In particular, the APOE4 genotype has been shown to increase the de novo synthesis^12^ or efflux of cholesterol^13^ in iPSC derived astrocytes, leading to the accumulation of cholesterol in the brain and decrease lysosomal function and elevate cytokine secretion from astrocytes^13^. Furthermore, APOE4 decreased uptake of extracellular fatty acids in iPSC-derived microglia^14^ or disrupted fatty acid clearance in iPSC-derived astrocytes, reducing the fatty acid transfer from neurons to astrocytes^15^. Recently, lipid droplet accumulation in APOE4 microglia have been linked to increased tau phosphorylation and neurotoxicity^16^, while APOE genotype–dependent neuronal lipofuscin accumulation and lysosomal lipid peroxidation have also been observed in iPSC-derived neurons^17^. We have previously shown increased population of glial-derived EVs resulting from neuroinflammation in AD patients’ brains^5,6,18^, suggesting that molecular structures in AD BDEVs could be highly impacted by glial lipid dysmetabolism mediated by APOE4 genotype. Given the lipid-rich nature of EV membranes, APOE-driven lipid remodeling may directly influence the composition and function of secreted EVs. In this study, we used multi-omics correlation analysis of EV-lipidome and proteome to identify complexed molecular signatures in E4/E4 BDEVs, which may promote increased neuronal EV uptake, potentially leading to exacerbated tau pathology in E4/E4 AD brains.

### APOE4/4 AD brain-derived EVs induce tau propagation and neuronal dysfunction

**Figure 1:**
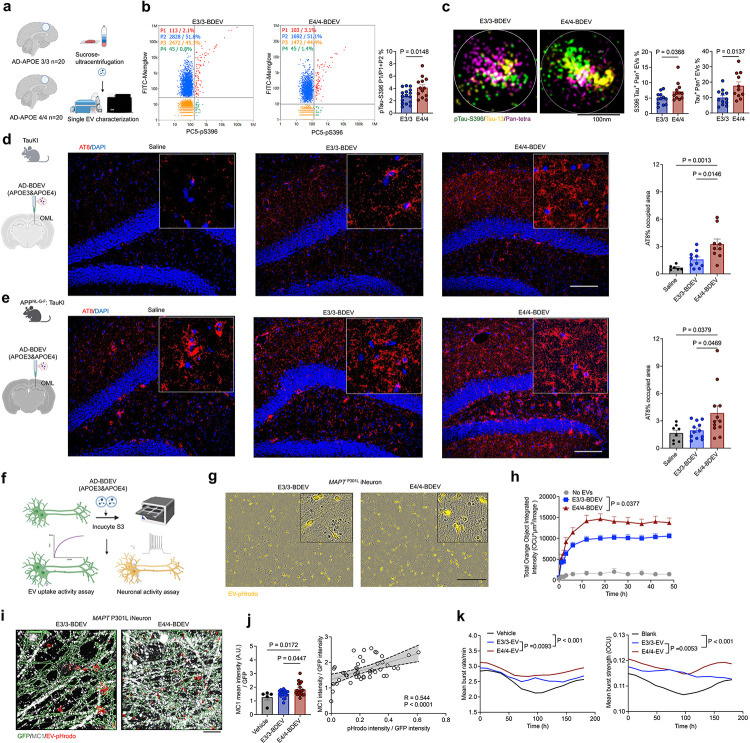
APOE4/4 BDEVs exacerbated tau pathology in aging humanized tau mice by enhancing neuronal uptake and activity **a,** Schematic of the BDEV isolation process from human brain tissue of AD patients carrying APOE3/3 or APOE4/4 using sucrose gradient ultracentrifugation, followed by single EV analysis with Nanoanalyzer and Nanoimager. **b,** NanoFlow cytometry analysis showed scatter plots of pTau-S396 conjugated with Alexa Fluor 647 (x-axis) versus MemGlow 488-labeled particles (y-axis) in BDEVs from APOE3/3 and APOE4/4, with quantification showing a significant increase of the P1 double-positive EV population in APOE4/4. **c**, Super-resolution microscopy images of BDEVs demonstrated co-localization of pTau-S396-Alex647/Tau-13-Alex594 with pan-tetraspanins-Alex 488 CD9, CD81, CD63 (scale bar: 100 nm). The percentage of triple-positive (pS396+/Tau+/pan-tetraspanins+) clusters and double-positive (Tau+/pan-tetraspanins+) clusters among total tetraspanins+ particles between APOE3/3 and APOE4/4 BDEVs. Each dot represents each case. **d**, Schematic of stereotaxic injection of APOE3/3 and APOE4/4 AD BDEVs (300 pg of tau) into the outer molecular layer (OML) of the hippocampus in Tau KI (**d**) and APP/Tau KI (**e**) mice at 10-month-old. **d-e**, Immunofluorescence staining for AT8 (red) and nuclei (blue) in the dentate gyrus (DG) regions of the hippocampus in Tau KI mice (**d**) and APP/Tau KI mice (**e**) after 3-month incubation. Scale bar: 100 μm. The AT8-positive area in the DG was quantified, with each dot representing the mean value from one animal (two sections per animal). BDEVs from three different donors were used per group and male and female mice were matched. **Tau KI:** Saline group (Male = 4, Female = 2), E3/3-BDEV group (Male = 4, Female = 7), E4/4-BDEV group (Male = 3, Female = 6); **APP/Tau KI**: Saline group (Male = 4, Female = 4), E3/3-BDEV group (Male = 7, Female = 5), E4/4-BDEV group (Male = 7, Female = 5). **f**, Schematic of the EV uptake and neuronal activity assay in iNeurons exposed to AD-BDEVs (APOE3/3 and APOE4/4), using the Incucyte S3 system. **g,** Representative images showed the uptake of APOE3/3-BDEVs and APOE E4/4-BDEVs (labeled with pHrodo-orange) by *MAPT*^P301L^ iNeurons. Scale bar: 200 μm. **h,** Quantification of the total orange object integrated intensity (OCU/μm^2^/image) over time in *MAPT*^P301L^ iNeurons (right) treated with APOE3/3-BDEVs (blue), APOE4/4-BDEVs (red), or only dye (grey). **i**, Representative 3D Imaris reconstructions showed GFP (green), misfolded tau MC1 (grey), and EV-pHrodo (red) in *MAPT*^P301L^ iNeurons treated with BDEVs (APOE3/3 and APOE4/4) or no EVs (vehicle). Scale bar: 5 μm. **j**, Quantified pHrodo mean intensity and MC1 mean intensity normalized to GFP in *MAPT*^P301L^ iNeurons. Correlation analysis between neuronal EV uptake and misfolded tau. At least three technical replicates. **k,** Quantitative changes in Ca^2+^ imaging signals were illustrated in a line graph, showing the mean burst rate per minute and mean burst strength (OCU) over time in WT iNeurons treated with BDEVs (APOE3/3 and APOE4/4) or PBS (vehicle). Significant differences were determined using ROC area under the curve analysis. BDEVs from three donors per group (APOE3/3 and APOE4/4) were analyzed, with technical replicates averaged. The Pearson correlation coefficient (r) and p-value are indicated. Statistical analyses were performed using a *t*-test and one-way ANOVA followed by Tukey’s post hoc test. Data are presented as mean ± SEM.

To investigate the effect of APOE phenotype on pathological roles of EVs in AD, we first isolated BDEVs from the temporal cortical regions of post-mortem brain tissues from APOE3/3 and APOE4/4 AD subjects, using sucrose gradient ultracentrifugation as we previously reported^19^. Twenty age- and sex-matched individuals with advanced Braak stages per group were selected (Fig. 1a, Supplementary Table 1 for demographic information). Transmission electron microscopic images of BDEVs demonstrated classical cup-shaped morphology and we found no significant differences in biophysical properties, including particle size, concentration, and protein yield between APOE3/3 and APOE4/4 BDEVs as determined by nanoparticle tracking analysis and Flow Nanoanalyzer (Extended Data Fig. 1a-j). Interestingly, single EV analysis using Flow Nanoanalyzer revealed a higher proportion of pS396 Tau^+^ EVs in APOE4/4 (4.14%) compared to APOE3/3 BDEVs (2.77%) although there were no significant changes in pT181 or Tau13 between the groups (Fig. 1b, Extended Data Fig. 2a-b). Direct stochastic optical reconstruction microscopy (dSTORM)-based super-resolution imaging of BDEVs by Nanoimager further validated these results by showing significantly increased pS396^+^ / total tau^+^ and a trend of increased pTau181 / total tau^+^ double-positive BDEVs in APOE4/4 compared to APOE3/3 group (Fig. 1c, Extended Data Fig. 2c). These data indicate an enrichment of pathogenic tau species in APOE4/4 AD BDEVs compared to APOE3/3 group.

To evaluate the effect of AD BDEVs on tau pathology development *in vivo*, we next stereotaxically injected APOE4/4 and 3/3 BDEVs (containing 300 pg tau in one μL) into the outer molecular layer (OML) of the dentate gyrus (DG) of human *MAPT* (Tau KI) and *APP*^NL-G-F^ : MAPT double knock-in (APP/Tau KI) mouse brains at 10 month-old (Fig. 1d, e, respectively). After a 3-month post-injection, immunofluorescence against AT8 antibody (for S205/T208 phosphorylated tau; p-tau) with Tau KI mouse brain tissues revealed a significant increase in AT8^+^ p-tau in the hippocampal DG, CA1 and CA3 fields after APOE4/4 BDEVs injection compared to APOE3/3 BDEVs injection and saline-injected control group (Fig. 1d and Extended Data Fig. 3a, b). There was no change in Alz50^+^ misfolded tau or glial activation as determined by intensity measurement of glial fibrillary acidic protein (GFAP)^+^ astrocytes, ionized calcium-binding adaptor molecule 1 (Iba1)^+^ microglia, and CD68^+^/Iba1^+^ microglia in the DG by both APOE BDEV injected groups compared to saline group in Tau KI mice (Extended Data Fig. 4a-d). Likewise, the brain tissues of APP/Tau KI mice injected with APOE4/4 BDEVs showed a significant increase in AT8^+^ p-tau in the DG, CA3 and CA1 compared to APOE3/3 group (Fig. 1e and Extended Data Fig. 3c, d). Notably, APOE4/4 BDEVs significantly promoted the accumulation of AT8^+^/fluorostyryl benzene (FSB)^+^ filamentous tau (neuritic plaque tau) compared to APOE3/3 in APP/Tau KI mice (Extended Data Fig. 3e, f). Additionally, there was significant increase in Alz50^+^ tau accumulation and CD68^+^ activated microglia in the DG induced by APOE4/4 BDEVs compared to saline group, which was absent in APOE3/3 group in APP/Tau KI mice (Extended Data Fig. 4e-h). These findings suggest a synergistic effect of APOE4/4 BDEVs on Ab deposition and tau propagation through phagocytic activation of microglia, underscoring their potential role in exacerbating AD pathology. To investigate the functional alteration in BDEVs recipient neurons, we assessed EV uptake by human induced pluripotent stem cell (iPSC)-derived neurons (iNeurons) and subsequent change in neuronal activity (Fig. 1f). Live imaging analysis using Incucyte S3 revealed increased uptake of APOE4/4 BDEVs, as indicated by enhanced internalization of pH-sensitive fluorescence (pHrodo)-labeled APOE4/4 BDEVs compared to APOE3/3 BDEVs in both WT and *MAPT*^P301L^ mutant iNeurons (Fig. 1g, h, Extended Data Fig. 5a and Supplementary Video 1–2). Notably, APOE4/4 BDEVs induced significantly higher MC1^+^ misfolded tau accumulation compared to vehicle and APOE3/3 BDEVs in *MAPT*^P301L^ neurons. Furthermore, there was a significant positive correlation between EV uptake and MC1 positivity in both WT and *MAPT*^P301L^ neurons, suggesting the possible tau seeding role by EVs (Fig. 1j and Extended Data Fig. 5b, c). When comparing the sex, APOE4/4 BDEVs from female AD cases (E4F-EVs) significantly increased their uptake by *MAPT*^P301L^ iNeurons, accumulation of pS396 tau level, and MC1 positivity in recipient *MAPT*^P301L^ iNeurons compared to the rest of the groups (Extended Data Fig. 5d-g). We next employed NeuroBurst-based Ca^2+^ imaging of iNeurons to understand how APOE genotype of BEDVs may affect recipient neuronal excitability. While we observed significantly higher neuronal burst rates and burst strength in both BDEVs compared to vehicle (PBS) administered WT neurons, APOE4/4 BDEVs significantly induced hyperactivity as determined by mean burst rate and disrupted network synchronization as determined by mean burst correlation in WT iNeurons compared to APOE3/3 BDEVs (Fig. 1k, Extended Data Fig. 5h and Supplementary Video 3–4). Taken together, these findings demonstrate that AD APOE4/4 BDEVs are more potent for promoting neuronal EV uptake, accumulation of misfolded tau and increase in neuronal burst strength and burst rate, highlighting their role in AD pathogenesis.

### Altered unsaturated fatty acid composition in APOE4/4 BDEVs

**Figure 2:**
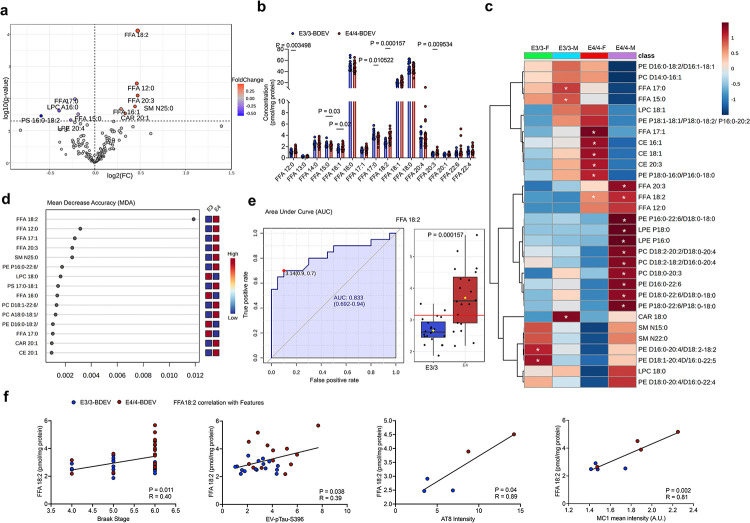
Lipid Profiling of APOE3 and APOE4 BDEVs from AD and their role in EV mediate tau pathology **a**, Volcano plot displayed differentially expressed lipids between APOE4/4-BDEVs and APOE3/3-BDEVs. Lipid species with significant changes (p < 0.05) are labeled. Red dots represent lipids upregulated in APOE4/4 BDEVs, while blue dots indicate those downregulated. Changes are color-coded based on fold change. **b,** Detailed analysis of free fatty acids (FFA) lipid molecule concentrations highlighted significant difference in specific FFAs between E3/3-BDEVs and E4/4-BDEVs. **c**, Hierarchical heatmap, clustered by lipid species displayed significantly altered lipids across four groups: APOE3/3 and APOE4/4 BDEVs from female and male patients. Changes are color-coded based on lipid expression intensity, with significant lipids marked by asterisks. **d,** Mean Decrease Accuracy (MDA) plot from random forest analysis identified the most important lipids for distinguishing between APOE3/3 and APOE4/4 BDEVs. FFA 18:2 is a key distinguishing lipid between the two groups. The colors range from high to low, indicating change of the value. **e**, Area Under Curve (AUC) analysis for FFA 18:2, illustrating its diagnostic potential in distinguishing between APOE3/3-BDEVs and E4/4-BDEVs in AD. AUC:0.833 (0.692–0.94). The box plot showed significantly higher levels of FFA 18:2 in APOE4/4 compared to APOE3/3. **f**, Correlation plots demonstrating the relationship between FFA 18:2 levels and various tau pathology markers, including Braak stage, EV-containing pTau-S396 levels (Fig. 1b), BDEV-induced AT8 intensity (Fig. 1e) and BDEV-induced MC1 mean intensity in *MAPT*^P301L^ iNeurons (Fig. 1j). The Pearson correlation coefficient (r) and p-value are indicated. Significant differences from direct comparisons between groups are indicated by asterisks p < 0.05.

We next performed comprehensive lipidomic profiling of AD APOE3/3 and APOE4/4 BDEVs (N=20 per group). A total of 173 lipid molecules were identified with notable enrichment in phosphatidylcholine (PC), phosphatidylethanolamine (PE), free fatty acids (FFAs), and cholesterol esters (CE), while mitochondrial CAR lipid contamination remain minimal (Extended Data Fig. 6a). Although overall lipid subclasses did not differ significantly between two groups (Extended Data Fig. 6b), APOE4/4 BDEVs exhibited significantly increased levels of free fatty acids (FFAs), particularly FFA 18:2, 16:1, 12:0, and 20:3, as well as sphingomyelin SM N25:0, while FFA 15:0, 17:0 and phosphatidylserine (PS) 16:0–18:2 and Lysophosphatidylcholine (LPC) A16:0 was significantly downregulated compared to APOE3/3 BDEVs (Fig. 2a, b, and Supplementary Table 2), suggesting that altered fatty acid metabolism in AD brain^20^ is reflected in the lipid composition of BDEVs. Sex-stratified analysis revealed that female APOE4/4 BDEVs exhibited higher CE levels (CE 16:1, 18:1, and 20:3) and reduced PE long chain fatty acids while male APOE4/4 BDEVs were enriched in saturated lysophosphatidylethanolamine (LPE 16:0 and 18:0) and PC with long-chain unsaturated fatty acids (Fig. 2c and Extended Data Fig. 6c, d), indicating that lipid composition of BDEVs is further influenced by sex. Notably, FFA 18:2 was elevated in both male and female APOE4/4 BDEVs and emerged as the top discriminator between the two genotypes with a higher predictive accuracy as determined by random forest model (Fig. 2d). Receiver operating characteristic (ROC) curve analysis further validated its diagnostic potential, with an area under the curve (AUC) of 0.833, pointing FFA 18:2, a polyunsaturated fatty acid (PUFA)^21^ as a potential biomarker for the early detection and stratification of APOE4 AD cases (Fig. 2e). Trait analysis shows significant positive correlations of FFA 18:2 levels in BDEVs with Braak stage, pS396 tau level in BDEVs, AT8^+^ tau pathology development after *in vivo* injection of BDEVs into APP/Tau KI mouse brains, and MC1^+^ tau accumulation in iNeurons after BDEV uptake *in vitro* (Fig. 2f). These findings suggest that dysregulated lipid composition in APOE4/4 BDEVs, especially enrichment of FFA 18:2, may drive tau pathology, potentially exacerbating neuroinflammation and disease progression in APOE4 AD cases.

### Lipid-protein network analysis identifies key molecules in EV-mediated tau pathways

**Figure 3:**
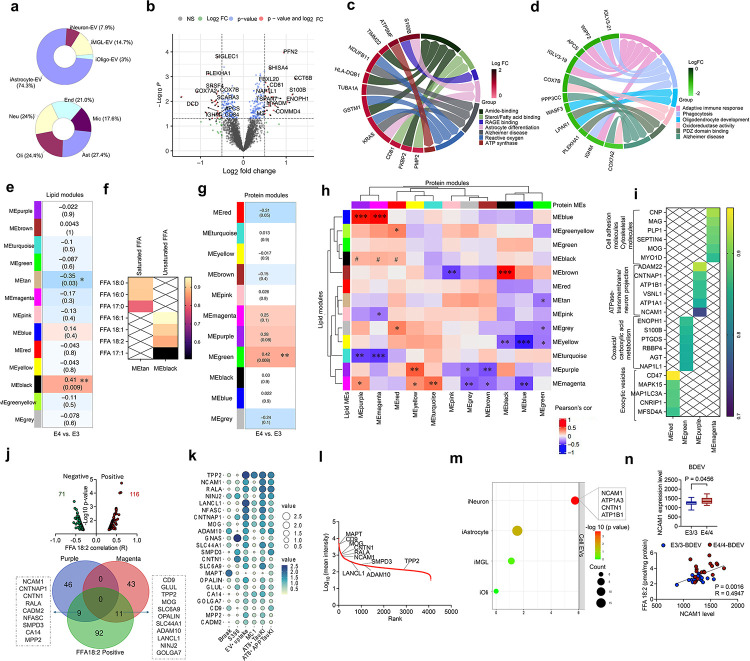
Network analysis discovered key lipid and protein modules in AD BDEV mediating tau pathology associated with APOE genotypes **a**, Pie charts represented the distribution of EV proteins across human neural cell type-specific EV dataset (You et al., 2022) and brain cell origin dataset (McKenzie et al., 2017), indicating the proportion of identified cell type-specific proteins. **b**, Volcano plot showed differentially expressed proteins identified between APOE4/4 and E3/3-BDEVs using DIA-MS. Proteins with statistically significant changes (p < 0.05) and a log2 fold change cutoff of ±0.5 were labeled. **c,** Chord diagram illustrated differentially expressed proteins in APOE4/4-BDEVs compared to APOE3/3-BDEVs, highlighting upregulated proteins (left) and downregulated proteins (right) along with pathways involved in biological functions. The connections represent protein-function relationships, with log_2_FC indicated by the color gradient. **e,** Heatmap showed the correlation between APOE genotypes (APOE4 vs. APOE3) and lipid modules identified using Weighted Gene Co-expression Network Analysis (WGCNA). The tan and black modules showed significant associations. Cell colors represent correlation coefficients: blue for negative and red for positive correlations. **f**, Heatmap showed key lipid molecules from GSEA, with the black module enriched in unsaturated fatty acids and the tan module in saturated fatty acids. Colors indicate enrichment scores. **g,** Heatmap displayed the correlation between APOE genotypes and protein modules identified using WGCNA. The green module showed significant associations with APOE genotype. The colors represent correlation coefficients. **h**, Cross-correlation heatmap showed the relationships between lipid and protein modules. Significant correlations were observed in two modules marked with (*) and a mild correlation between lipid (black) and protein modules (purple, r = 0.2; magenta, r = 0.22; red, r = 0.19 marked with #). *p < 0.05, **p < 0.01, ***p < 0.01. **i**, Heatmap displayed the enrichment of molecular function and biological processes associated with key proteins from different modules based on GSEA. Key proteins for each module and their involvement in pathways were listed on the left. The enrichment scores were represented by the color gradient. **j**, Volcano plot highlighted proteins positively and negatively correlated with FFA18:2 (top); The Venn diagram illustrated the overlap of protein modules (purple and magenta) with the FFA 18:2 positive protein pattern, with shared protein name listed in the box. **k**, Bubble plot showed the associations between key proteins and tau pathology alternations including Braak stage, EV pTau-S396 levels (Fig. 1b), EV uptake, MC1 intensity (Fig. 1j), and BDEV-induced AT8 intensity (Fig. 1e). Bubble size and color indicate the −log10(p-value) of Pearson correlations. **l,** Line plot ranked BDEV proteomics protein intensities, highlighting key proteins. **m**, Cell-type origin analysis of EV showed the enrichment of the purple and magenta module proteins across different cell types based on the human neural cell type-EV dataset (You et al., 2022). iNeurons have the highest enrichment score, with the top four genes highlighted. Bubble color and size indicate rank and expression level. **n**, NCAM1 levels from BDEV proteomics were compared between APOE3/3 and APOE4/4 groups and were found to be positively correlated with FFA18:2. T-test and Pearson correlation were conducted.

To further elucidate molecular mechanisms underlying APOE4 BDEVs-driven tau pathology, we performed proteomic analysis using data-independent acquisition (DIA) mass spectrometry from the same cohort samples from proteome, which identified total of 4027 unique proteins (Supplementary Table 3). Integration of the dataset with iPSC-derived neuronal and glial EV^18^ and human brain transcriptomic database^22^ confirmed that these BDEVs are primarily derived from astrocytes, with lesser contributions from neurons, microglia, oligodendrocytes, and endothelial cells (Fig. 3a). Volcano plot analysis identified significantly altered proteins in APOE4/4 BDEVs compared to APOE3/3 BDEVs (p < 0.05, |log_2_FC| > 0.5) (Fig. 3b and Supplementary Table 3). Upregulated proteins in APOE4/4 BDEVs were primarily associated with sterol/fatty acid binding and astrocyte differentiation (Fig. 3c), while downregulated genes were linked to adaptive immune response and phagocytosis (Fig. 3d), suggesting potential functional differences of the EV-secreting cells. Notably, proteins in female APOE4/4 BDEVs were enriched in astrocyte- and microglia-derived EV markers, cholesterol biosynthesis, oxidative phosphorylation/cell adhesion, and protein translation (Extended Data Fig. 7a-c). Proteins in male APOE4/4 BDEVs showed enrichment in astrocyte- and neuron-derived EV markers, metabolism, and extracellular space / tubulin pathways (Extended Data Fig. 7a-c). These findings suggested that AD BDEVs possess unique proteomic signatures in APOE genotype and sex-dependent manner, which may influence the biological function of BDEVs.

Leveraging the data obtained from the same samples for the lipid and protein compositions, we integrated both datasets using an orthogonal approach to better understand the distinct biological outcomes at a molecular level. We therefore performed two-dimensional Weighted Gene Co-expression Network Analysis (2D-WGCNA) using identified 173 lipids and 4027 proteins from BEDV samples and found 13 lipid modules and 11 protein modules correlating each other (Fig. 3e, g). Among 13 lipid modules, two modules were significantly differentially expressed in APOE4/4 compared to APOE3/3 BDEVs: MEblack, enriched in unsaturated FFAs, including FFA18:2 as top molecules, showing positive association with APOE4, and MEtan, enriched in saturated FFAs, showing negative association with APOE4 (Fig. 3e, f, Extended Data Fig. 8a and Supplementary Table 4). On the other hand, MEgreen (oxoacid/carboxylic acid metabolism) showed significant positive association with APOE4 among 11 protein modules (Fig. 3g, Extended Data Fig. 8b and Supplementary Table 4). We observed strong protein interactions with two APOE4-enriched black and tan lipid modules. MEblack lipid moedule was moderately correlated with protein modules MEmagenta (enhanced cell adhesion), MEpurple (ATPase transmembrane activity/neuron projection), and MEred (exocytic vesicle transport pathways). In contrast, MEtan lipid module exhibited a significant inverse correlation with MEgreen protein module (Fig. 3h). Furthermore, APOE4-enriched MEgreen protein module was significantly negatively associated with lipid modules MEtan, MEgray (PC-unsaturated fatty acids) and MEyellow (PC-saturated and monounsaturated fatty acids and SM) lipid modules. Additionally, there are strong interactions between EV lipid and proteins independent of APOE genotype; MEbrown lipids (cardiolipin, CL) were associated with MEpink and MEblack protein modules linked to mitochondrial function. MEblue and MEturquoise (both containing PE-unsaturated fatty acids) lipid modules are positively or negatively correlated with MEpurple or MEmagenta protein module, respectfully. MEyellow lipid module was significantly negatively correlated with both MEblue and MEblack protein modules mainly involved in mitochondrial transport and activity (Fig. 3h, i and Supplementary Table 5). These data indicate corelated dysregulation in lipid metabolism and protein biological pathways.

To uncover key molecules underlying the APOE4/4 BDEV-mediated tau pathology progression, we analyzed 12 biological pathways significantly positively correlated with FFA18:2, an upregulated FFA in APOE4/4 vs. APOE3/3 and specific protein profiles, which ranked cell adhesion molecules pathways as the most enriched pathway (Extended Data Fig. 8c, d). The volcano plot analysis of correlated proteins with FFA 18:2 shows that 116 and 71 proteins were positively and negatively correlated with FFA 18:2 (Fig. 3j), respectively. The Venn diagram depicted 11 overlapped proteins in MEmagenta and 9 overlapped proteins in MEpurple modules with 116 positively correlated proteins with FFA 18:2 (Fig. 3j). Further correlation analysis revealed significant association between 20 selected proteins and tau pathological features from *in vitro* and *in vivo* study, highlighting the top-ranked proteins such as TPP2, NCAM1, and RALA (Fig. 3k). A protein rank plot of proteomics showed NCAM1 and RALA were much more abundant than the top-ranked TPP2 (Fig. 3i). Notably, NCAM1 was enriched in EVs from iNeurons, elevated in APOE44 BDEVs, and correlated with FFA18:2 compared to RALA (Fig. 3m, n and Extended Data Fig. 8e), suggesting its specific regulation by FFA18:2.

### FFA18:2 enhanced NCAM1 expression and amplified tau propagation via EVs

**Figure 4:**
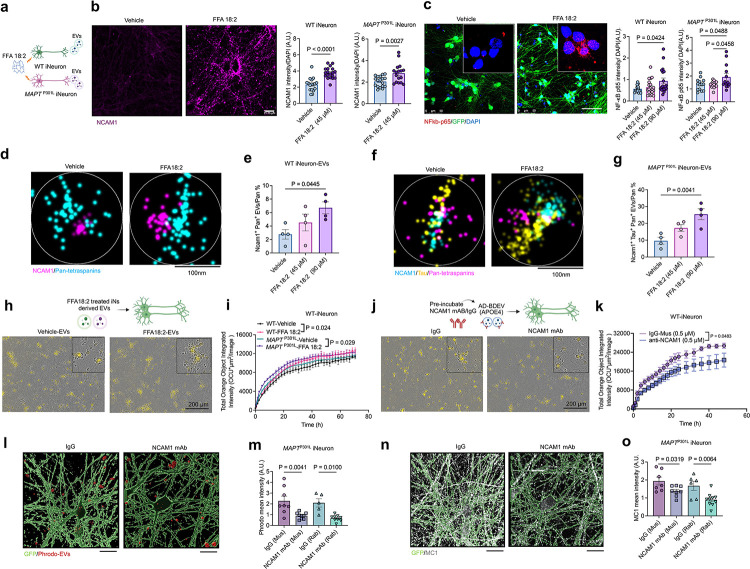
The effect of Linoleic Acid FFA 18:2 on adhesion molecules and its role in tau propagation via EVs. a, Schematic of the experiment to investigate the effects of FFA 18:2 on cell adhesion molecules and EV associated tau inclusion in WT and *MAPT*^P301L^ iNeurons. b, Surface NCAM1 expression in WT and *MAPT*^P301L^ iNeurons following FFA 18:2 treatment. Representative images of surface NCAM1(magenta) and quantification of immunofluorescence intensity normalized to DAPI (nuclear stain). Scale bar: 10 μm. c, NFκB-p65 expression in iNeurons following FFA 18:2 treatment. Representative images and quantification of NFκB-p65 (red) immunofluorescence intensity normalized to DAPI. Scale bar: 50 μm. d, Nanoimaging of EVs isolated from WT iNeurons treated with 90 μM FFA 18:2, showed co-localization of NCAM1 (magenta) and pan-tetraspanin markers (cyan) at a single EV. Scale bar: 100 nm. e, Quantification of NCAM1^+^ EVs as a percentage of total Pan-tetraspanin^+^ EVs in WT iNeurons with different FFA 18:2 concentrations. f, Nanoimaging of EVs isolated from *MAPT*^P301L^ iNeurons treated with FFA 18:2 (90 μM), showed NCAM1 (cyan), tau (yellow), and Pan-tetraspanin (magenta) co-localized in a single EV (Scale bar: 100 nm). g, Quantification of NCAM1^+^/Tau^+^ EVs as a percentage of total Pan-tetraspanin+ EVs in *MAPT*^P301L^ iNeurons treated with different concentrations of FFA 18:2. Each dot represented the average value from each technical replicates of EV sample. h, WT iNeurons were treated with EVs isolated from vehicle- or FFA18:2-treated iNeurons. Neuronal EV uptake was monitored and quantified using the Incucyte S3 system. Representative images showed EV-pHrodo (yellow) uptake within iNeurons. i, Line graph indicated that EVs from FFA 18:2-treated iNeurons had higher uptake activity, shown by increased orange signal intensity. j, iNeurons underwent an EV uptake assay with AD-BDEVs (APOE4/4) labeled with pHrodo dye, under NCAM1 antibody blockade. Representative images showed BDEV (yellow) uptake in WT iNeurons treated with control IgG or anti-NCAM1 monoclonal antibody (mAb). k, AD-BDEV uptake in WT iNeurons was quantified over time and compared between IgG and NCAM1 mAb treatments. l-0, 3D-representative images showed GFP-positive WT (l) and *MAPT*^P301L^ iNeurons (n) co-stained with pHrodo-EVs (red) and MC1 (grey) after treatment with NCAM1 mAbs or their IgG controls. Scale bar: 5 μm. (m, o) Quantification showed the mean intensities of pHrodo and MC1 in WT and *MAPT*^P301L^ iNeurons treated with control IgG or NCAM1 mAbs. Each dot represented a ROI from IPSCs from different technical replicates. Data are presented as mean ± SEM. One-Way ANOVA followed with Tukey’s *post hoc*.

FFA18:2, an essential omega-6 fatty acid implicated in induction of inflammation and cell adhesion molecules^21,23^, was investigated for its role in NCAM1 expression and tau propagation via EVs *in vitro*. Immunofluorescence confirmed NCAM1 localization in MAP2^+^ neurons in human brain tissue (Extended Data Fig. 9a). To test the biological effect of FFA 18:2 *in vitro*, iNeurons were treated with FFA18:2 at varying concentrations for 24 h, followed by collection of cells and EV for the biological analyses (Fig. 4a). Interestingly, FFA18:2 treatment induced surface expression of NCAM1 in both WT and *MAPT*^P301L^ iNeurons (Fig. 4b), although total NCAM1 levels in the cell lysates were unchanged (Extended Data Fig. 9b, c). To evaluate the induction of inflammation^23^, FFA18:2 treatment significantly enhanced nuclear localization of NFκB p65, a key indicator of inflammation in both WT and *MAPT*^P301L^ iNeurons (Fig. 4c). To directly examine cargo loading of NCAM1 to EVs, we quantified NCAM1^+^ EVs isolated from FFA18:2-treated WT iNeurons by Nanoimager (Fig. 4d,e) and Nanoanalyzer (Extended Data Fig. 9d,e). There was a significant, dose-dependent increase in NCAM1^+^ EVs under FFA18:2 treatment (Fig. 4d, e and Extended Data Fig. 9d, e), despite no change in total pan-tetraspanin^+^ EV numbers (Extended Data Fig. 9f, g). To further assess the effect of FFA18:2 treatment on tau loading to EVs, we treated *MAPT*^P301L^ iNeurons with FFA18:2 and found that it increased the number of NCAM^+^/Tau^+^ EVs in a dose-dependent manner as determined by Nanoimager (Fig. 4f, g). Moreover, EVs derived from FFA18:2-treatediNeurons exhibited their enhanced uptake by recipient iNeurons, with *MAPT*^P301L^ iNeurons-derived EVs showing a particularly strong effect (Fig. 4h, i and Extended Data Fig. 9h, i), suggesting that FFA18:2 treatment facilitate neuronal internalization of secreted EVs. To determine whether NCAM1 is a key mediator of the neuronal EV uptake, pHrodo-labeled APOE4/4 BDEVs were preincubated with anti-NCAM1 or control monoclonal antibodies (mAbs) and applied to iNeurons for live imaging of EV uptake (Fig. 4j). Anti-NCAM1 mAb pre-treatment significantly reduced BDEV uptake compared to IgG controls (Fig. 4k). For further validation, two different anti-NCAM1 antibodies also reduced BDEV uptake compared with IgG treated EVs in *MAPT*^P301L^ iNeurons (Fig. 4l, m), which is accompanied by a significant reduction in MC1^+^ tau accumulation in *MAPT*^P301L^ iNeurons (Fig. 4n, o). These findings demonstrate NCAM1 as a key mediator of FFA18:2-induced tau loading to EVs and their uptake by neurons. Therapeutic targeting of NCAM1 could provide a strategy to disrupt EV-mediated tau propagation, particularly in APOE4 carriers, thereby mitigating tau pathology in recipient neurons.

### NCAM1 monoclonal antibody attenuated tau propagation and neuroinflammation

**Figure 5:**
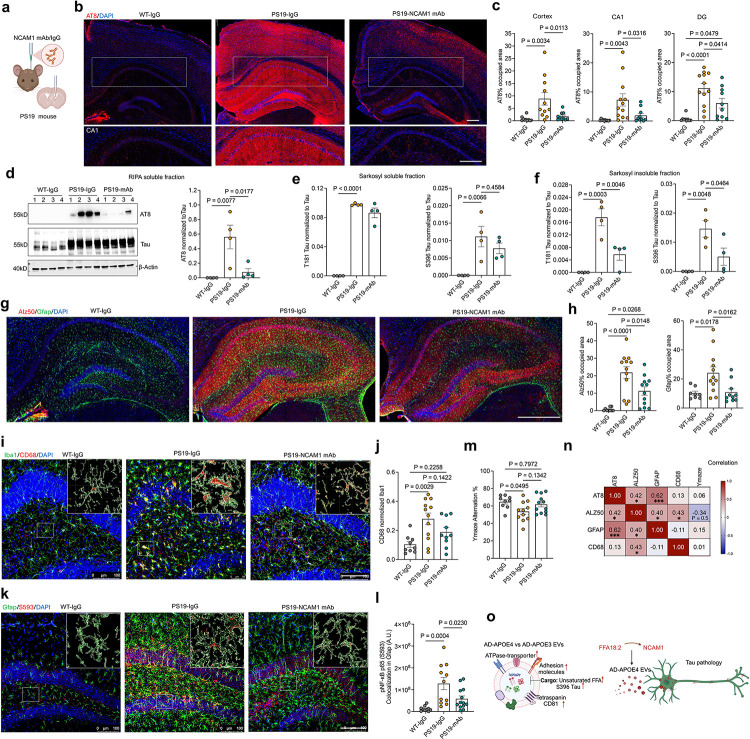
NCAM1 monoclonal antibody reduces tau pathology and neuroinflammation in PS19 mice. **a**, Schematic of intracerebroventricular (ICV) injection with NCAM1 monoclonal antibody (mAb) or IgG control into PS19 mouse brains at 6.5 months of age. Mouse brain tissue were assessed one month later. b, Representative fluorescence images of AT8+ phosphorylated tau (red) and DAPI (blue) in the cortex and hippocampus, with an enlarged view of the CA1 region from WT (IgG) and PS19 (IgG, mAb) mice. Scale bar: 500 μm. c, Quantification showed NCAM1 mAb-treated PS19 mice displayed significantly reduced AT8+ tau accumulation compared to PS19 IgG-treated mice, exhibiting a pattern similar to WT-IgG controls in cortex, CA1 and dentate gyrus (DG). Male and female mice were matched d, Western blot analysis of AT8+ phosphorylated and total tau in RIPA-soluble hippocampus lysates from WT-IgG, PS19-IgG, and PS19-NCAM1 mAb-treated mice. Quantification showed a significant reduction in the ratio of AT8+ tau to total tau in the NCAM1 mAb-treated PS19 group. e, f, ELISA-based quantification of phosphorylated tau (T181 and S396 epitopes) in sarkosyl-soluble (S2) and insoluble (P2) tau fractions across three groups. NCAM1 mAb treatment significantly reduced tau phosphorylation at T181 and S396 epitopes in the P2 fractions. g, Representative fluorescence images of Alz50+ misfolded tau (red), Gfap (green) and DAPI (blue) in the hippocampus.Scale bar: 500 μm. h, Quantification indicated NCAM1 mAb treatment significantly reduced Alz50+ tau burden and GFAP astrocytosis in hippocampal DG of PS19 mice. i, Representative fluorescence images of CD68+ (red) microglial activation within Iba1+ (green) microglia in the DG of WT and PS19 mice treated with IgG and mAb with a large view showing Imaris rendering. Scale bar: 100 μm. j, Quantification showed that PS19 mice exhibited increased CD68 intensity compared to WT and NCAM1 mAb treatment modestly reduced CD68. k, Representative fluorescence images of pNF-κB p65 (S536) and GFAP (green) across the three groups with a large view showing Imaris rendering. Scale bar: 100 μm. l, Quantification showed increased pNF-κB activation in PS19 mice compared to WT, which was significantly reduced by NCAM1 mAb treatment. i, Y-maze behavioral analysis showed NCAM1 mAb treatment increased the percentage of alternations in PS19 mice compared to IgG-treated controls. m, Correlation matrix showed associations between tau pathology (AT8 and Alz50 in the DG data), neuroinflammation markers (GFAP, CD68), and behavioral performance. A strong positive correlation was indicated by asterisks. WT-IgG (Male = 5, Female = 4), PS19-IgG (Male = 7, Female = 4), PS19-NCAM1 (Male =8, Female = 4). n, Schematic of the proposed mechanism of AD-APOE4/4 BDEV contributes to AD pathology. Data are presented as mean ± SEM. One-Way ANOVA followed with Tukey’s *post hoc*.

To evaluate the therapeutic efficacy of NCAM1 mAb on tauopathy development *in vivo*, we utilized the human *MAPT*^P301S^ transgenic mice (PS19 line), a well-established model for tauopathy^24^ and exhibits increased FFA18:2 levels in the brain compared to wild type (WT) mice^25^. We observed notable enrichment of NCAM1 in BDEVs from PS19 mice compared to WT controls (Extended Data Fig. 10a). At 6.5 months of age, PS19 mice received intracerebroventricular (ICV) injections of NCAM1 mAb (5 μg in 10 μL DPBS), while control PS19 and WT mice received ICV injection of control IgG (Fig. 5a). Strikingly, after one-month incubation, histopathological analysis revealed significant reduction in AT8^+^ tau accumulation in the cortex and hippocampus, including DG, CA1, CA2 and CA3 regions in anti-NCAM1 mAb treated PS19 mice compared to control IgG treated group (Fig. 5b, c and Extended Data Fig. 10b). In agreement with these immunofluorescence data results, biochemical analyses confirmed significant reduction in AT8^+^ tau / total tau ratio in RIPA-soluble lysates and phosphorylated tau (S396, T181) in sarkosyl-insoluble fractions in anti-NCAM1 mAb treated PS19 mice compared to the control IgG group while there was no significant difference between anti-NCAM1 and control mAb groups in sarkosyl-soluble fraction (Fig. 5d–f and Extended Data Fig. 10c). Alz50^+^ misfolded tau accumulation was also markedly decreased in the DG of NCAM1 mAb-treated PS19 mice (Fig. 5g, h). Beyond tau pathology, NCAM1 mAb treatment significantly reduced the number of GFAP^+^ astrocytes, indicative of suppressed astrogliosis (Fig. 5g, h), along with a trend of decrease in CD68^+^ phagocytic microglia (Fig. 5i, j). Since we observed activation of NFκB by FFA18:2 treatment of neurons *in vitro*, we examined the level of pS593 NFkB, its activation marker. NCAM1 mAb treatment suppressed the expression of pS593 NFkB^+^ GFAP^+^ astrocytes (Fig. 5k, l), suggesting the effect of NCAM1 on the neuroinflammatory responses via NFkB signaling. Finally, behavioral studies of these animals demonstrate improved spatial working memory as determined by increased alternation in Y-maze in NCAM1 mAb-treated PS19 mice (Fig. 5m). Correlation analyses of the outcome measures from the same animals pointed that tau pathology markers AT8 and Alz50 were significantly correlated, and both tau markers were strongly associated with GFAP. Additionally, Alz50 positively correlated with CD68 and negatively with Y-maze performance (Fig. 5n). Taken together, our study demonstrates that: AD APOE4/4 BDEVs were enriched in pathogenic tau, FFA18:2 and NCAM1, driving tau aggregation and neuroinflammation. Treatment of iNeurons with FFA18:2 upregulates NCAM1, recruits NCAM1 and tau to EVs, enhancing their neuronal uptake and tau seeding *in vitro*. Strikingly, central treatment of PS19 mice with NACM1 mAb is beneficial for disrupting the BDEV-mediated tau propagation and neuroinflammation, which is significantly associated with cognitive impairment (Fig. 5o). Our study demonstrates that targeting NCAM1 represents a promising strategy to mitigate tauopathy in AD.

## Discussion

Here, we performed a comprehensive biological characterization of BDEVs from late-onset AD cases with APOE3/3 and APOE4/4 genotypes, with a particular focus on their role in tau propagation and neuroinflammation. We reported that APOE4/4 BDEVs contain higher levels of phosphorylated tau (pS396), significantly enhance internalization and accelerate tau pathology in both *in vivo* using aged Tau KI and APP/Tau KI mice and *in vitro* using WT and *MAPT*^P301L^ iNeurons. Lipidomic and proteomic profiling of human AD BDEVs revealed distinct molecular signatures associated with APOE4/4 BDEVs, including elevated levels of unsaturated fatty acids, particularly the pro-inflammatory FFA18:2, and a strong enrichment of cell adhesion molecules linked to NFκB signaling activation. Notably, NCAM1 emerged as a key molecule within this network, and its targeted inhibition effectively attenuated EV-mediated tau pathology development *in vitro* and *in vivo*, suggesting NCAM1 as a potential therapeutic target for mitigating disease progression, particularly in APOE4 carriers.

APOE4 is the most dominant genetic risk factor for AD, increasing susceptibility up to 15-fold in e4 homozygous individuals^26^. Multiple studies have demonstrated that APOE4 exacerbates tau pathology, with evidence from human, cellular, and animal models showing its role in promoting tau hyperphosphorylation and aggregation. APOE4 carriers exhibit elevated tau-PET signals independent of amyloid burden^27^, increased pathological tau accumulation in iPSC-derived cerebral organoids^28^ and exacerbated tau pathology in animal models^29^. Studies in APOE4 KI:PS19 mice demonstrate increased tau spread in APOE4 KI compared to APOE3 KI background^29^. However, the role of APOE in BDEV-mediated tau transmission remains poorly understood.

BDEVs from AD patients contains filamentous tau^7^ and facilitate tau propagation more effectively than soluble tau aggregates, inducing synaptic dysfunction in recipient cells^30^. Our data demonstrated that APOE4 BDEVs contain a higher proportion of pS396-Tau^+^ EVs and higher tau load per EV compared to APOE3 BDEVs, suggesting that APOE4 BDEVs carry pS396-Tau, a marker of neurofibrillary tangle maturation and disease progression^5^. In APP/Tau KI mice, APOE4/4 BDEVs facilitated the formation of tau filaments, accompanied by increased activation of phagocytic microglia. *In vitro*, APOE4/4 BDEVs exhibited increased neuronal uptake and induced tau misfolding, particularly in *MAPT*^P301L^ iNeurons, which exacerbated the pathology process. Mechanistically, treatment of iNeurons with APOE4/4 BDEVs induced pronounced hyperactivity as determined by the mean burst strength and rate. This is well supported by the observation that APOE4 KI mice exhibit excessive neuronal activation and impaired homeostatic plasticity^31^.

Alterations in EV lipid profiles have been implicated in disease progression^32^. In AD BDEVs, significant alterations in glycerophospholipid, sphingolipid, and PUFA levels were observed compared to controls^8^. Our lipidomic analysis of AD brain samples, with a focus on APOE genotype, revealed notable differences in EV lipidome between APOE3/3 and APOE4/4 brain sample. Notably, APOE4/4 BDEVs exhibited significantly higher levels of FFA18:2 (linoleic acid), an omega-6 PUFA primarily derived from dietary sources. Although linoleic acid constitutes less than 2% of total brain fatty acids, up to 59% of dietary linoleic acid is capable of crossing the blood-brain barrier^33^. Preclinical studies suggest that excessive dietary intake of linoleic acid may exacerbate neuroinflammation and promote the production of oxidized lipid metabolites^34^. Additionally, linoleic acid has been implicated in modulating seizure susceptibility, ischemia-induced brain injury, and neurotransmission via its oxidized metabolites^35,36^. APOE4 impairs FA storage in neuronal lipid droplets, leading to FFA buildup and metabolic dysfunction in conjunction with astrocyte lipid coupling, underscoring the need for timely clearance of FFAs to prevent neurodegeneration^15,37^. Furthermore, we observed higher levels of SM N25:0, a long-chain sphingomyelin, in APOE4/4 BDEVs. This enrichment is indicative of enhanced sphingomyelinase activity known to promote EV biogenesis at the plasma membrane and within endocytic compartments.

Interestingly, female APOE4 BDEVs showed higher levels of cholesterol esters, such as CE16:1 and CE18:1. These polyunsaturated CE species carried by APOE4 vesicles may contribute neuronal lipofuscin accumulation and lysosomal lipid peroxidation, ultimately promoting intercellular cholesterol buildup^17^. Excess cholesterol stored in lipid droplets is cytotoxic, as cholesterol cannot be enzymatically degraded within the cell^38^. In contrast, male APOE4 BDEVs exhibited increased levels of LPE, PC, and PE, indicating lipid dysmetabolism in broader species. These lipid alterations influence membrane fluidity and dynamic modulation of lipid raft-mediated trafficking, thereby affecting cargo loading into EVs and EV internalization by recipient cells, which was supported our observation of enhanced APOE4/4 BDEV uptake by iNeurons. The interaction between lipids and proteins plays a critical role in these processes, with proteins such as Alix binding to lysobisphosphatidic acid to regulate vesicle budding and fusion^39^. In addition, our proteomic analysis revealed an elevated levels of proteins in cholesterol and fatty acid binding including PMP2 (lipid transport and myelination) and FKBP2 (protein folding and trafficking). Female APOE4/4 BDEVs showed enrichment in cholesterol-binding functions and lipoprotein particles that link with cholesterol ester enrichment, particularly in elevated PLP1 protein which is subtype derived gene linked to AD pathology^40^. Characterization of these molecules will further advance our understanding of lipid and protein-based BDEV cargo molecule development.

The protein and lipid compositions of EVs were analyzed using integrated 2D-WGCNA, revealing key interactions that regulate EV functions. Notably, unsaturated fatty acids positively correlated with exocytic vesicle activity, particularly reflected by enhanced expression of cell adhesion molecules and ATPase transporters, while saturated fatty acids negatively correlated with protein modules related to oxoacid metabolism. This indicated that APOE4 reduced the sequestration of fatty acids in neuronal lipid droplets, elevating free fatty acid levels, particularly FFA18:2, which promotes lipid peroxidation, ROS production, and oxidative stress, as supported by our WGCNA analysis identifying increased oxyacid-related proteins and ATP transport in neuronal pathways^15,41^. Previous reports suggest that FFA18:2, an unsaturated FFA, promotes inflammation and increase expression of cell adhesion molecules^23,42^. This 2D-WGCNA indicated that the certain lipid species and protein components play a crucial role in EV cargo delivery, modulating EV uptake, tau transfer, and propagation. Among them, NCAM1 identified as a key modulator of neuron-neuron adhesion, with its enrichment on the EV surface^43^, potentially drives inflammation through the unsaturated fatty acid pathway. NCAM1 has previously been shown increased expression in murine reactive astrocytes to response CNS injury^44^. Our finding suggested that elevated NCAM1 levels in response to FFA18:2 in AD APOE4/4 BDEV facilitate enhanced EV internalization, exacerbating pathological protein transport. Given that NCAM1 regulates EV uptake through cell adhesion mechanisms and NCAM1-mimetic peptides have anti-inflammatory properties^45^, NCAM1-targeting strategies is a promising avenue for tauopathy intervention, particular in APOE4 carriers.

In terms of the limitation of the study, our study focused on AD cases with APOE3 and APOE4 genotypes; incorporating healthy controls in future research could provide deeper insights into allele-specific contributions. Additionally, as most cases included were from the late Braak stages, expanding to earlier disease stages could help reveal genotype-driven differences before advanced pathology develops. Humanized APOE4/4 tau models will be beneficial to further assess the therapeutic potential of NCAM1 targeting in tau pathology. Moreover, long-term monoclonal antibody therapy may offer greater potential for improving cognitive function.

Collectively, these results highlight the functional interplay between lipid and protein components of APOE4 BDEVs, revealing a mechanistic link between adhesion molecules, fatty acid metabolism, and EV-mediated tau pathology. Targeting NCAM1 could reduce EV uptake by recipient neurons, mitigating the EV-mediated tau propagation and neuroinflammation, thereby halting disease progression in APOE4 AD cases.

## Methods Section

This study was exempt from human studies as determined by the Mayo Clinic IRB. Animal studies were approved by Mayo Clinic Institutional Animal Care and Use Committee.

### Human brain sample acquisitions

The postmortem brain tissues (cortical temporal lobe) used in this study were obtained from individuals diagnosed with Alzheimer’s Disease (AD) from the Mayo Clinic Brain Bank. We included 20 cases with the APOE3/3 genotype and 20 cases with the APOE4/4 genotype. All brain tissue-derived extracellular vesicles (BDEVs) were isolated for characterization, biological assays, Omic studies, and functional validation. The demographic information for all cases, including age, sex, genotype, postmortem interval (PMI), and diagnosis were listed in supplemental Table 1.

### Isolation of EVs from AD human brain by sucrose gradient ultracentrifugation

The brain EVs were isolated according to our published method^19^. Briefly, 1.0 g of frozen cortical brain tissue was sliced into small pieces with a razor blade and dissociated in Hibernate-E medium (Cat# A1247601, Thermo Fisher Scientific) at 75 U/mL collagenase type 3 (Worthington # CLS-3, S8P18814) at 37°C for 15 minutes. Protease and phosphatase inhibitors (Cat# 78442, Thermo Scientific) and 7 mL of ice-cold Hibernate-E solution were then added to stop the digestion. After passing through a 40-μm mesh filter (Cat# 22-363-547, Thermo Fisher Scientific) to remove large fragments, the supernatant was centrifuged at 2000 × *g* for 15 minutes, followed by 5000 × *g* for 20 min at 4°C. The supernatant was then filtered through a 0.45-μm polyethersulfone membrane filter (Cat# SLHPM33RS, Millipore) and ultracentrifuged at 100,000 × *g* for 70 minutes at 4°C using a SW41Ti rotor (Optima-XE SW41, Beckman Coulter). The pellet was resuspended in 2 mL of ice-cold 0.475 M sucrose (Cat# S5-3, Thermo Fisher Scientific) in double-filtered PBS (dfPBS, MF-Millipore^®^ Membrane Filter, 0.22 μm pore size). This suspension was overlaid on five sucrose cushions (2 mL each of 2.0 M, 1.5 M, 1 M, 0.825 M, and 0.65 M in dfPBS) to enrich EV pellets by centrifugation at 200,000 × *g* for 20 hours at 4°C. The targeted EV fractions were from layer V and VI with fresh 8 mL cold dfPBS, and ultracentrifuged at 100,000 × *g* for 70 minutes at 4°C. The final pellets were resuspended in 45 μL dfPBS as the purified EVs.

### hiPSC-neurons (iNeurons)

Rapid induction of iPSC into human excitatory neurons was performed via a doxycycline-inducible neurogenin-2 (NGN2) system using our published protocol^18^. Briefly, to initiate NGN2 induction, doxycycline was added to NGN2 iPSCs at a final concentration of 2 μg/mL in fresh media consisting of KnockOut DMEM (Cat# 10829018), KnockOut Serum Replacement (Cat# 10828010), MEM-NEAA (Cat# 11-140-050), Glutamax (Cat# 35050061), and β-mercaptoethanol (Cat# 21985023, all from Invitrogen). On Day2, puromycin was added at 5 μg/mL for selection. On Day4, cells were replated on poly-ornithine (10 μg/mL; Cat# P4957, Sigma-Aldrich) and mouse laminin (5 μg/mL; Cat# 23017015, Invitrogen) co-coated 6-well plate (1 × 10^6^ cells/well) in media consisting of Neurobasal (Cat# 21103049, Gibco), B27 (Cat# 17504044, Gibco), Glutamax, 20% dextrose (Cat# G8769, Sigma-Aldrich), MEM-NEAA supplemented with 10 ng/mL BDNF (Cat# 450-02), GDNF (Cat# 450-10) and CNTF (Cat# 450-13, all from PeproTech). In this study, we used two iPSC-derived neuronal lines bearing *APOE3/3* genotype: one is derived from a healthy individual (WT iNeurons) and the other one is derived from a frontotemporal dementia patient carrying the *MAPT*^P301L^ mutation (*MAPT*^P301L^ iNeurons) collected by Mayo iPSC core facility.

### FFA 18:2 treatment on iNeuron

iNeurons were treated with FFA 18:2, specifically linoleic acid (Cat # L1012-1G, Sigma), which was prepared as a 1M stock solution. FFA 18:2 was diluted to final concentrations of 4.5 μM, 45 μM, and 90 μM in B27-containing medium without serum. iNeurons were incubated for 24 hours, then washed and replaced with fresh neuron culture medium (EV pellet removal) for an additional 4 d. The culture medium was collected, and cells were either lysed or fixed for downstream experiments.

### Isolation of EVs from iNeuron medium

According to the protocol^46^with minor modifications, To enrich EVs, the medium was collected and subjected to sequential centrifugation: first at 300 × *g* for 10 min, followed by 2,000 × *g* for 10 min, then at 5,000 × *g* for 30 minutes at 4°C, and finally at 100,000 × *g* for 70 min. After removing the supernatant, the pellets of EV were washed with dfPBS and centrifuged again at 100,000 × *g* for 70 min. The final pellet was resuspended in 15 μL of dfPBS and used for NanoFCM or Nanoimager-based analyses.

### EV characterization

#### Transmission electron microscopy (TEM)

The morphology of isolated EVs was analyzed using a transmission electron microscope according to our published method^19^. A volume of 5 μL EV sample was adsorbed onto a carbon-coated grid (Electron Microscopy Sciences) for 5 min. Excess liquid was removed with filter paper, and the grid was washed briefly with water to eliminate residual phosphate or salt. It was then stained with 0.75% uranyl formate (Cat# 22451 EMS) for 15 s, after which excess stain was removed, and the grids were examined using a JEM-1400Flash electron microscope (JEOL).

#### Nanoparticle tracking analysis (NTA)

The size distribution and concentration of EVs were measured using a NanoSight NS300 instrument (Malvern, Worcestershire, UK). All the EV samples were diluted in dfPBS (1:700) and injected into the sample carrier cell. The instrument tracked and sized particles using Brownian motion, recording four 30s videos at a detection temperature of 22.5°C ± 0.5°C with setting level 5 and camera level 13. Video images were analyzed with Nanosight NTA 3.4 software (Malvern Panalytical Inc.), yielding size mode (nm), size (nm) and concentration (particles/mL) data.

#### Flow Nanoanalyzer (Nano-FCM)

We used Nano-flow cytometry to measure different fluorescence-labeled EV populations according to the protocol (10.3791/64020). EV samples were diluted to a concentration of 1–2 × 10^10^ particles/mL in dfPBS. A 4.5 μL aliquot of the diluted EV samples was incubated with 1.5 μL of a mix of fluorescent antibodies and incubated overnight at 4°C in the dark. We tested unstained EVs and samples with only antibodies as controls. For analysis, 1 μL of the labeled sample was diluted 1:50–1:100 in dfPBS to achieve an event number range of 4000–6000. Samples were acquired under 1.0 kPa sampling pressure for 1 minute each. EV subpopulations were analyzed using the auto-threshold. We included MemGlow 488 fluorogenic membrane dye (Cat# MG01-02, Cytoskeleton) at 40 nM to label the total membrane structure particles and other fluorescent antibodies included anti-Tau 13 (1:40, Cat# MMS-520R, BioLegend) conjugated with Alexa Fluor 647 (Cat #ab269823, Abcam). anti-pTau Ser396 Alexa Fluor 647 (1:30, Cat# 829006, BioLegend), anti-pTau T181 Alexa Fluor 647 (1:15, Cat# 846608, BioLegend) for human brain EVs; anti-NCAM1 Alexa Fluor 647 (1:10, Cat#563443, BD Biosciences) for iNeuron-derived EVs.

#### Super-resolution microscopy analysis

We utilized super-resolution microscopy to capture single molecules in EVs using a temperature-controlled Nanoimager S Mark II microscope from ONI (Oxford Nanoimaging, Oxford, UK), equipped with a 100x, 1.4NA oil immersion objective. We followed an established protocol^19,47^ with minor modifications. The cover glasses were coated with 8-methoxypyrene-1,3,6-trisulfonic acid, trisodium salt (MPTS, Cat# M395, Fisher Scientific), dissolved at 6% in ethanol, for 30 minutes. The glasses were then incubated with Sulfo-GMBS (10 μM in PBS, Cat# PI22324, Fisher Scientific) for another 30 min. After treatment, the glasses were incubated with human TIM4/Human Fc Chimera recombinant protein (1 mg/mL, Cat# 081-10261, Fujifilm) overnight at 4°C. A 2 μL volume of the diluted EV sample (2 × 10^10^ particles/mL) was mixed with 2 μL blocking buffer and incubated with antibodies overnight at 4°C. The fluorescent antibodies in this study used included anti-pTau S396 Alexa Fluor 647 (1:40, Cat# 829006, BioLegend), anti-pTau T181 Alexa Fluor 647 (1:40, Cat#8466008, BioLegend,), anti-Tau 13 (1:40, Cat# MMS-520R, BioLegend) conjugated with Alexa Fluor 594 (Cat# ab269822, Abcam), and anti-NCAM1 Alexa Fluor 647 (1:1, Cat#563443, BD Biosciences). For detecting tau inclusion in EVs, 0.01% Triton will be used. To normalize total EVs, a pan-tetraspanin antibody cocktail was used, consisting of anti-human CD9-FITC (HI9a, Cat# 312104), anti-human CD81-FITC (5A6, Cat# 349504), and anti-human CD63-FITC (H5C6, Cat# 353006; all from BioLegend). Only the dye without the EV sample was used to test quality and dilution for the fluorescence antibody. The stained EVs were then treated with 1 μL of CaCl_2_ (1 μM) and pipetted onto the pre-coated cover glasses for attachment over 1 hour. Following PBS washes, the fixation buffer (4% PFA + 4% sucrose) was applied, followed by 10 μL of mixed ONI B-Cubed Imaging Buffer (Alfatest, Rome, Italy) for EV imaging. Two channels (2000 frames per channel) (647 and 488 nm) and three channels (1000, 1500, and 1000 frames for 647, 562, and 488 nm, respectively) were acquired sequentially at 30 Hz in total internal reflection fluorescence (TIRF) mode. All images were analyzed using algorithms developed by ONI via their CODI website platform (https://alto.codi.bio/project/). Filtering and drift correction were performed using NimOS software following with temporal groups. The BD-Scan clustering tool was used to merge channels, with the bin size set to 20 and the maximum radius (r) set to 150 nm.

#### Protein Extraction and Concentration

Brain tissue and enriched EV fractions from APOE3 and APOE4, as well as cells after washing with PBS, were lysed in cold RIPA buffer (25 mM Tris-HCl pH 7.6, 150 mM NaCl, 1% NP-40, 1% sodium deoxycholate, 0.1% SDS; Cat# 89900, Thermo Fisher Scientific) supplemented with a protease and phosphatase inhibitor mixture (Cat# 78442, Thermo Scientific) and sonicated for 2 minutes at 4°C. Protein concentrations were determined using Micro Bicinchoninic Acid (microBCA) kit (Cat# 23235 Pierce, Thermo Fisher Scientific) under incubation at 60°C for 30 min. The absorbance was measured using a Biotek Synergy Mx plate reader at 562 nm.

### Western Blot and Elisa

Equivalent amounts (5 μg) of total brain and EV proteins, as well as 15 μg of protein from iNeuron samples, were measured using the microBCA kit and denatured in Laemmli sample buffer (Cat# 1610737, Bio-Rad). The samples were then loaded onto SDS-PAGE gels (Cat# 4561096, Bio-Rad) and electro-transferred to 0.45 μm nitrocellulose membranes (Cat# 1620115, Bio-Rad). The membranes were blocked with 5% non-fat milk (Cat# 9999S, Cell Signaling Technology) for 1 hour and incubated overnight at 4°C with primary antibodies. The following primary antibodies were used for western blot: anti-CD9 (1:500, #CBL162, Millipore), anti-Cytochrome C (CytoC, 1:200, #11940S, Cell Signaling Technology), anti-FLOT1 (1:500, #610820, BD Biosciences), anti-Annexin A2 (ANXA2, 1:200, #ab178677, Abcam), anti-NCAM1 (1:500, #MA5-11563, Thermo Fisher Scientific), anti-pSer202/pSer205 tau (AT8, 1:1000, Cat# MN1020, Thermo Fisher Scientific) and human tau (1:1000, Cat# A0024, DAKO) and anti-beta actin (1:1000, #sc-130065, Santa Cruz). The membranes were then incubated with horseradish peroxidase (HRP)-labeled secondary antibodies (anti-mouse IgG, HRP-linked antibody, #7076S; anti-rabbit IgG, HRP-linked antibody, #7074S, Cell Signaling Technology) for 1 h. Immunoreactivity was detected using enhanced chemiluminescence solutions (Millipore, WBKLS0100). For silver staining, protein gels were fixed and stained following the manufacturer’s instructions (Cat# 24612, Thermo Fisher Scientific). For the quantification analysis, a rectangular area was selected enclosing the bands of interest, and the intensity of each band was measured from the area showing the most intense signal using ImageJ. The signal intensities of all protein bands were normalized by the β-actin band. The levels of phosphorylated tau proteins (pT181 and pS396) and total human tau in iNeuron lysates, as well as in the S2 and P2 fractions isolated from mouse hippocampal tissue, were quantified using ELISA (Invitrogen; Cat. #KHO0631, KHB7031, and KHB0041, respectively) according to the manufacturer’s protocol.

### Immunohistochemistry and Immunofluorescence

Immunofluorescence was employed to characterize postmortem human and mouse brains, as well as iNeuron samples. Mouse brains were collected following PBS perfusion, post-fixed in 4% PFA for 48 h, and then transferred to 30% sucrose in PBS at 4°C. The tissues were subsequently embedded in O.C.T. compound (Thermo Fisher Scientific). Coronal cryosections (25 μm in thickness) encompassing the hippocampal region were prepared using the free-floating staining method, with two sections per mouse utilized for each primary antibody immunofluorescence analysis. Antigen retrieval using sodium citrate pH 8.0 buffer was performed by incubating sections at 85°C for 20 min, followed by two rinses with PBS. Sections were then blocked and permeabilized using 5% goat serum (Cat# 16210-064, Thermo Fisher Scientific), 5% bovine serum albumin (BSA; A7906, Sigma-Aldrich), and 0.3% Triton^™^ X-100 in TBS for 1.5 h. The sections were incubated overnight at 4°C with the following primary antibodies diluted in a solution containing 2.5% BSA, and 0.15% Triton in TBS: anti-pSer202/pSer205 tau (AT8, 1:200, Cat# MN1020, Thermo Fisher Scientific), anti-misfolded tau (Alz50, 1:50, provided by Dr. Peter Davies), anti-Clec7a (1:300, Cat #mabg-mdect, Invitrogen), anti-GFAP (1:2000, Cat# Z033429, Agilent), anti-Iba1 (1:1000, Cat # 019-19741, Wako Chemicals), anti-CD68 (1:500, Cat# 137001, BioLegend), anti-NF-κB p65 (1:500, Cat# 8242S, Cell Signaling Technology).

For human brain sections, slides were heated at 60°C for 30 min, followed by deparaffinization with xylene and a series of ethanol washes. Antigen retrieval was performed using 1× Co-Detection Target Retrieval Reagent (Advanced Cell Diagnostics, USA) for 15 min at 98°C, according to the manufacturer’s protocol^43^. The primary antibodies used were: anti-NCAM1 (1:300, Cat#14255-1-AP, ProteinTech), anti-MAP2 (1:200, Cat#13-1500, Thermo Fisher Scientific).

For cultured cells, the cells were washed three times with 1× Dulbecco’s PBS, fixed in 4% PFA plus 4% sucrose at 37°C for 15 min, and then blocked at room temperature for 1 h. Surface protein NCAM1 was stained without permeabilization, while 0.3% Triton was applied for other proteins. The following primary antibodies were used: anti-misfolded tau (MC1, 1:100, provided by Dr. Peter Davies), anti-NCAM1 (1:500, Cat#14255-1-AP, Proteintech), anti-NFκB (1:50, Cat# G2210, Santa Cruz Biotechnology). Detecting surface NCAM1 were without any permeability treatment. Alexa Fluor secondary antibodies (1:1000, Life Technologies) were used for detection, with the conjugation performed at room temperature (25°C) for 1 hour. DAPI (4′,6-diamidino-2-phenylindole; 1:2500, Thermo Fisher Scientific, D1306) was used to label cell nuclei. All images were captured using a Nikon deconvolution wide-field epifluorescence system (Nikon Instruments) or a Leica SP8 confocal microscope (Leica).

#### Confocal image processing and quantification

Images were acquired using a Leica SP8 confocal microscope (Leica) in confocal mode with a 20 × objective and 1.0 optical zoom, at a pinhole setting of 1.0 airy unit. Confocal image stacks were captured at a resolution of 1024 × 1024 pixels, with a system-optimized z-interval of 0.5 μm. Following image acquisition, all images were processed using adaptive LIGHTNING deconvolution (Leica) to enhance image clarity and resolution. For iNeuron imaging, MC1, and pHrodo-EV signals were acquired using zoom level 3, with 5–10 cells included in each ROI. For NCAM1 and NFκB staining, zoom level 1 was used to capture 15–20 cells per ROI. For AT8+ signal analysis, ImageJ software was employed with the particle size range set between 10–1000 for quantification. Colocalization analysis of AT8 with FSB or Clec7a was performed using Imaris software, utilizing a mask to quantify AT8 signal colocalization. The signals for GFAP, Alz50, CD68, Iba1 and pNFκB were quantified using the Imaris surface function, with auto-thresholds set to ensure accurate quantification. Additionally, microglial and NF-κB activation were assessed by masking and surface rendering of the CD68 signal within Iba1^+^ cells and NFκB within GFAP^+^ cells, respectively. Final data analysis was performed with Excel (Microsoft) and Prism 10 (GraphPad).

#### APOE AD EV and iNeuron EV Labeling with Phrodo Dye and PKH26

EVs were labeled using either a pHrodo dye (#P36600, Therom Fisher Scientific) or the lipophilic red fluorescent dye PKH26 (#MINI26-1KT, Sigma-Aldrich), following modified protocols to ensure optimal labeling efficiency and minimal free dye contamination^48^. Phrodo Dye Labeling method: 20 μg of EV proteins were mixed with 0.5 μL of pHrodo red dye with a concentration of 10.6 mM) and incubated at room temperature for one hour; PKH26 Labeling method: 6 μg of EV samples were mixed with 0.8 μl of PKH26 dye in 60 μl of Diluent C, achieving a final concentration of 5 μM. The mixture was incubated for 10 min at 37°C.

For the two labeling methods, to terminate the labeling reaction and remove free dye, 350 μL of cold double filtered PBS was added. The labeled EVs were then transferred to an Amicon Ultra-0.5 Centrifugal Filter Unit with a 100 kDa membrane (#UFC510096, EMD Millipore) and centrifuged at 14,000 × *g* for 5 min to enrich the labeled EVs. The final concentration of labeled EVs was adjusted to 0.8 μg/100 μL BDEVs or positive particle numbers (iNEV) for subsequent neuronal EV uptake assays.

#### Labeled ADEV Uptake by iNeurons Using the IncuCyte System

Phrodo or PKH26-labeled APOE3/3-BDEVs, APOE4/4-BDEVs (0.4 μg / 50 μL), and iNeuron-derived EVs (1:200 ratio of cell intensity to EVs) were prepared. For the negative control, an equivalent volume of dye-only solution was used. The labeled EVs or controls were added to each well containing iNeuron (WT or *MAPT*^P301L^) at a density of 25,000 cells/well (in a 96 well plate) in EV-depleted iNeuron medium. For the antibody inhibition experiment, a final concentration of 0.5 μM of specific antibodies, including anti-NCAM1 (mouse species, #MA5-11563, Thermo Fisher Scientific; rabbit species, #14255-1-AP, Proteintech), or isotype controls (mouse IgG, #MAB002, R&D Systems; rabbit IgG, #ab210849, Abcam), were pre-incubated with the labeled ADEVs at 37°C for 30 min. After pre-treatment, the EVs were added to DIV18 iNeuron (WT or *MAPT*^P301L^) at a concentration of 0.4 μg / 50 μL. The cells were placed in the IncuCyte ZOOM system (Essen BioScience, Ann Arbor, MI), housed in a humidity chamber at 37°C with 5% CO₂, as described previously^48^. Images were captured from 9 fields per well every two hours over a total period of 48 h. Image processing and analysis were conducted using IncuCyte S3 software. The orange fluorescence signal in each treatment was monitored over time, serving as an indicator of EV uptake efficiency. The data were quantified by measuring the total integrated intensity of the orange fluorescence (OCU μM^2^/Image) across the imaging period.

#### Calcium Imaging Analysis by IncuCyte S3

25K iNeurons / well in a 96-well plate (WT or *MAPT*^P301L^) at DIV10 were transfected with 1.5 μL of NeuroBurst Orange Lentivirus (Cat. #4736, Sartorius) in 50 μL of iNeuron medium. The cells were incubated with the lentivirus for one day. After 24 h, the lentivirus-containing medium was removed, and fresh iNeuron medium was added. The neurons were then cultured for an additional 7 d before EV treatment. 0.5 μg / 50 μL of unlabeled BDEVs were added to the transfected iNeurons, and the cells were incubated for 8 days. Half of medium was refreshed every 4 d during this incubation period. Live-cell imaging was performed using the IncuCyte S3 system, with fluorescence images captured every 12 h over the 8-day period. This imaging setup allowed for the tracking of the temporal dynamics of calcium signaling in response to EV treatment. The analysis focused on key metrics implemented in the IncuCyte system, including burst strength (OCU), burst rate, and correlation.

#### EV Protein Cleanup, Digestion, and LC-MS/MS analysis

The purified EV proteins were reduced with 5 mM dithiothreitol (DTT) for 30 min, alkylated with 10 mM iodoacetimide (IAA) for 30 min in the dark, and quenched with 10 mM DTT for 20 min, all at room temperature (RT), then subjected to paramagnetic beads cleanup and trypsin digestion as described previously^49^. Briefly, a 1:1 mix of two types (hydrophilic (Sera-Mag Speed Beads, GE Life Sciences, CAT# 45152105050250) and hydrophobic (Sera-Mag Speed Beads, GE Life Sciences, CAT# 65152105050250)) of beads were added to EV proteins at a bead to protein ratio of 5:1 (w/w). 100% ethanol was added to the mixture to reach a final concentration of 50%, and then incubated on a shaker for 10 min at 1000 rpm at R/T. The supernatant was discarded after resting the samples on a magnetic rack, and the beads were washed 3 times with 80% ethanol. The digestion was carried out by adding digestion solution (10 ng/uL trypsin in 10 mM ammonium bicarbonate) to the beads (with protein bounded) at a 1:50 protease to protein ratio, at 37 °C on a shaker overnight. The digested peptides were transferred into a new tube after resting the samples on the magnet. The beads were washed with 60% acetonitrile and subsequently 5% formic acid, the wash solutions were combined into the same tube and vacuum dried before analysis. About 50 ng peptides from each sample were loaded on a C18 column and analyzed on the Bruker timsTOF SCP mass spectrometer in a 60 min gradient, with the diaPASEF approach.

#### Protein Identification and Quantification

The raw timsTOF data (. d folders) were imported into DIA-NN (version 1.8) and searched against the *in-silico* library generated with downloaded human (proteome ID: UP000005640) protein sequences from the UniProt database. The search results were filtered with precursor q value of <0.01 at the library level and protein group q value of <0.01. For quantification, the precursor quantities were first obtained by DIA-NN through summing the intensities of the top six fragments (ranked by their library intensities) for each precursor. Precursors corresponding to unique proteins were then used for protein-level quantification, and the intensities of protein groups were obtained using the MaxLFQ algorithm implemented in the *iq* r package^50^. Cross-run normalization was then performed based on the median protein intensity of each sample.

#### The purified EV samples for the Lipidomics

Lipid species was analyzed using multidimensional mass spectrometry-based shotgun lipidomics approach^51^. In brief, each extracellular vesicle sample was homogenized, and an equivalent of 0.09 mg protein homogenate was then added to a glass tube along with a pre-mixed lipid internal standard. Lipid extraction was performed using a modified Bligh and Dyer procedure^52^. The lipid extract was dispersed in chloroform:methanol (1:1, v:v) at a ratio of 400 μL/mg protein for storage. For shotgun lipidomics, the lipid extract was further diluted to a total lipid concentration of ~2 pmol/μL. The mass spectrometric analysis was performed on a triple quadrupole mass spectrometer (TSQ Altis, Thermo Fisher Scientific, San Jose, CA) and a hybrid quadrupole-Orbitrap mass spectrometer (Q-Exactive, Thermo Fisher Scientific, San Jose, CA), both equipped with an automated nanospray ion source device (TriVersa NanoMate, Advion Bioscience Ltd., Ithaca, NY) as described previously^53^.The data processing and analysis was performed based on the principles of shotgun lipidomics such as ion peak selection, baseline correction, isotope effect correction, etc.^51,54,55^. The final lipicomics result were normalized to the protein content (nmol/mg protein).

#### Mouse maintenance

Humanized *APP*^NL-G-F^ and *MAPT*^hTau/hTau^ (Tau KI) mice were obtained from RIKEN (Drs. TC Saido and T. Satio)^56^ and cross-bred to generate *APP*^NL-G-F/NL-G-F^:*MAPT*^hTau/hTau^ double knock-in homozygous (APP/Tau KI mice). Tau P301S (Line PS19) expressing human 1N4R FTDP-17-linked P301S mutation was obtained from Jackson Laboratory (B6;C3-Tg(Prnp-MAPT*P301S) PS19Vle/J, #008169). Wild-type (WT) strain B6C3F1 was used to maintain P301S transgene hemizygotes. Animals were housed as groups in regular light/dark cycles with free access to food and water, and welfare-related assessments were carried out before and after the surgery. Mice were randomly allocated to experimental groups, and variability was assessed based on the body weight. Both APP/Tau KI and Tau KI mice were used for intracerebral inoculation of human BDEVs. PS19 mice were used for intracerebroventricular (ICV) administration of anti-NCAM1 antibodies. All animal procedures followed the guidelines of the NIH Guide for the Care and Use of Laboratory Animals and were approved by the Mayo Clinic Institutional Animal Care and Use Committee.

#### Stereotaxic surgery

We perform the surgery according to our published protocol^6^. APP/Tau KI and Tau KI (10.5-month-old) were deeply anaesthetized with isoflurane and secured in a mouse stereotaxic frame (David Kopf Instruments) equipped with robotic stereotaxic injection system (Drill and Injection Robot, Neurostar). The animals were unilaterally inoculated with brain-derived EVs from AD APOE3/3 or APOE4/4 brains in the dorsal hippocampal OML (AP −2.18 mm; ML 1.13 mm; DV −1.9 mm from the skull) using a 10-μL Hamilton syringe. Each injection site received 1.0 μL of inoculum, containing 300 pg tau / μL for EV samples or saline.

For the NCAM1-blocking BDEV incubation, BDEV with APOE4/(300 pg tau / 1 μL) were incubated with either an anti-human NCAM1 monoclonal antibody or an isotype-matched IgG control (both 5 μM, 0.5 μL, azide-free) for 30 min **at 37°C**. Following incubation, a total volume of 1.5 μL was stereotaxically injected into the hippocampus of Tau KI mice. Mice were then maintained for a post-injection period of 3 months for further analysis.

For the therapeutic evaluation of NCAM1 in PS19 mice (6.5–7 months old), animals were anesthetized with continuous isoflurane and secured in a stereotaxic frame (Kopf). A burr hole was drilled at the following coordinates: AP +0.3 mm; ML +1.0 mm; DV −3.0 mm (relative to bregma, right hemisphere) according to our published protocol^57^. A Hamilton syringe (Cat #7653-01) with a 10-μL needle (Hamilton, Cat #7758-04) was used to deliver 5 μg of NCAM1 mAb or control IgG diluted in 10 μL of PBS at an infusion rate of 1 μL/min using an injection pump (World Precision Instruments). Post-surgery, mice were monitored and allowed to recover in a temperature-controlled environment. Following the one-month incubation, the mice will be conducted mouse behaviors and tau pathology assay.

### Mouse behaviors

#### Y-Maze Test

Spontaneous alternation behavior in the Y-maze was used to assess short-term spatial working memory. The maze consisted of three arms (each 40 cm long, 13 cm high, 3 cm wide at the bottom, and 10 cm wide at the top), converging at equal angles. Mice were placed at the center of the maze, facing one of the three arms (A, B, or C in sequence), and were allowed to explore freely for 10 minutes. Arm entries were defined as the complete entry of the mouse’s body into an arm. Alternation behavior was recorded using ANY-maze software (Stoelting Co). The spontaneous alternation percentage was calculated using the following formula: Spontaneous Alternation (%) = (Number of Alternations/Total Arm Entries–2) ×100, where an alternation was defined as consecutive entries into all three arms in a non-repetitive sequence (e.g., ABC).

#### Biochemical Sequential Extraction of Sarkosyl-Soluble and Insoluble Fractions

To enrich tau oligomers and fibrils, hippocampal tissue was subjected to sequential biochemical extraction, as previously described^57^, with minor modifications. Briefly, each hippocampal sample was homogenized in TBS buffer supplemented with a protease and phosphatase inhibitor mixture (Cat. #78442, Thermo Scientific). The homogenate was centrifuged at **55,000 rpm** (**Beckman Coulter TL-Series rotor**) for **30 minutes at 4°C**, and the resulting supernatant was collected as the **soluble (S1) fraction**. The pellet was then re-homogenized in **salt/sucrose buffer** (0.8 M NaCl, 10% sucrose, 10 mM Tris-HCl, pH 7.4, 1 mM EGTA) supplemented with protease and phosphatase inhibitors and centrifuged again at **55,000 rpm for 30 minutes at 4°C**. The resulting pellets were discarded, and the supernatants were incubated with **1% sarkosyl** (Sigma, Cat. #61747) for **one h at 37°C** with continuous agitation. The mixtures were ultracentrifuged at **55,000 rpm for one h at 4°C**. The supernatants were collected as **S2 fractions**, while the pellets (**P2 fractions**) were resuspended in **8 M guanidine buffer** and solubilized for **3 hours at room temperature** with continuous agitation. The solubilized **P2 fractions** were then diluted in RIPA buffer (Cat# 89900, Thermo Fisher Scientific) supplemented with protease and phosphatase inhibitors for subsequent ELISA analysis.

#### Bioinformatics Analysis (Lipidomics and Proteomics)

Lipid molecule data were exported into MetaboAnalyst 6.0 (https://www.metaboanalyst.ca/) for bioinformatic analysis. Significant lipid expression thresholds were defined using a criterion of p-value <0.05 (−log10(p-value) >1.3) to compare two genotypes (APOE3/3 vs. APOE4/4) or gender (Female vs Male), which were used to create a volcano plot. In addition, 3D-principal component analysis (PCA), heatmaps, and mean decrease accuracy (MDA) were generated using the statistical analysis module within the software. Biomarker analysis was conducted using a receiver operating characteristic (ROC) curve-based approach to identify potential biomarkers and evaluate their performance.

For proteomics analysis, the criteria for the volcano plot was set to a p-value <0.05 (−log_10_(p-value) >1.3) and fold change (|log_2_FC| > 0.5). Differentially expressed proteins (DEPs) were identified and exported for functional pathway analyses, including Gene Ontology analysis and Kyoto Encyclopedia of Genes and Genomes (KEGG) pathway analysis, using the DAVID pathway tool (https://david.ncifcrf.gov/). The DEPs were mapped to biological processes using STRING software and ranked based on fold change values (Szklarczyk et al., 2019). Cell-specific enrichment analysis was performed using the Hypergeometric Test, based on our published dataset^18^. Visualization of Venn diagrams, pathway bubble plots, and correlation bubble plots was performed using the R programming language (version 4.1.0).

#### Weighted Correlation Network Analysis (WGCNA) of Brain-Derived EV Lipidome and Proteome

Weighted correlation network analysis (WGCNA) was utilized to define co-expression networks of lipids and proteins within BDEVs. The analysis was conducted using R (version 3.6.3) following previously established protocols. The smallest power, 14, was selected to achieve a scale-free topology (R^2^ = 0.9).

For both lipidome and proteome data, normalized abundance values were input into the WGCNA::blockwiseModules() function to construct the networks. The proteins and lipids were hierarchically clustered by calculating the topological overlap (TO) using the bicor correlation function. Module assignments were determined using dynamic tree-cutting, with the following parameters specified: soft threshold power (beta) = 14, deepSplit = 4, minimum module size = 2, merge cut height = 0.2, signed network with partitioning around medoids respecting the dendrogram, and a reassignment threshold of p < 0.05. The module eigengenes (MEs), which represent the first principal component of each module, were computed to encapsulate the most representative abundance value for each module, explaining the covariance of all proteins or lipids within the module. Pearson correlations were computed to relate each protein or lipid to their respective MEs, and these correlations were also assessed for significance.

### Statistical analysis

Experimenters were blinded to the genotypes during testing. Data are presented as the mean ± standard error of the mean (SEM). Statistical analyses were performed using GraphPad Prism (version 10.0). Comparisons between two groups were analyzed for significance using Student’s unpaired. For comparisons among three or more groups, ordinary one-way ANOVA with Tukey’s post hoc test was used. Correlation analyses were conducted using Pearson or Spearman tests based on the data distribution. Area under the curve (AUC) values were derived from receiver operating characteristic (ROC) analysis. Statistical significance was defined as a p-value < 0.05. P-values in functional enrichment analyses were adjusted for multiple comparisons using the false discovery rate (FDR) correction with the Benjamini-Hochberg method where applicable. Outliners could be identified and removed using Q = 1%.
